# Risk of adenocarcinomas of the oesophagus and gastric cardia in patients hospitalized for asthma

**DOI:** 10.1054/bjoc.2001.2094

**Published:** 2001-09-01

**Authors:** W Ye, W-H Chow, J Lagergren, P Boffetta, G Boman, H-O Adami, O Nyrén

**Affiliations:** Department of Medical Epidemiology, Karolinska Institute Box 281, Stockholm, SE, 171 77, Sweden; Division of Cancer Epidemiology and Genetics, National Cancer Institute, Bethesda, MD, 20892, USA; Department of Medical Sciences; Respiratory Medicine and Allergology, Uppsala University, Uppsala, SE-75185, Sweden; Unit of Environmental Cancer Epidemiology, International Agency for Research on Cancer, Lyon, 69008, France; Department of Epidemiology, Harvard School of Public Health, Boston, MA, 02115, USA

**Keywords:** asthma, gastro-oesophageal reflux, adenocarcinoma of the oesophagus and gastric cardia

## Abstract

In the first cohort study of the question we followed 92 986 (42 663 men and 50 323 women) adult patients hospitalized for asthma in Sweden from 1965 to 1994 for an average of 8.5 years to evaluate their risk of oesophageal and gastric cardia adenocarcinoma. Standardized incidence ratio (SIR) adjusted for gender, age and calendar year was used to estimate relative risk, using the Swedish nationwide cancer incidence rates as reference. Asthmatic patients overall had a moderately elevated risk for oesophageal adenocarcinoma (SIR = 1.5, 95% confidence interval CI, 0.9–2.5) and gastric cardia cancer (SIR = 1.4, 95% CI, 1.0–1.9). However, the excess risks were largely confined to asthmatic patients who also had a discharge record of gastro-oesophageal reflux (SIR = 7.5, 95% CI, 1.6–22.0 and SIR = 7.1, 95% CI, 3.1–14.0, respectively). No significant excess risk for oesophageal squamous-cell carcinoma or distal stomach cancer was observed. In conclusion, asthma is associated with a moderately elevated risk of developing oesophageal or gastric cardia adenocarcinoma. Special clinical vigilance vis-à-vis gastro-esophageal cancers seems unwarranted in asthmatic patients, but may be appropriate in those with clinically manifest gastro-oesophageal reflux.   http://www.bjcancer.com © 2001 Cancer Research Campaign


*British Journal of Cancer* (2001) **85**(9), 1317–1321
© 2001 Cancer Research Campaign
doi: 10.1054/ bjoc.2001.2094, available online at http://www.idealibrary.com on IDEAL

http://www.bjcancer.com

### Risk of adenocarcinomas of the oesophagus and gastric cardia in patients hospitalized for asthma

**W Ye[1], W-H Chow[2], J Lagergren[1], P Boffetta[1,4], G Boman[3], H-O Adami[1,5] and O Nyrén[1]**

[1]Department of Medical Epidemiology, Karolinska Institute Box 281, SE 171 77, Stockholm, Sweden; [2]Division of Cancer Epidemiology and Genetics, National Cancer Institute, Bethesda, MD 20892, USA; [3]Department of Medical Sciences; Respiratory Medicine and Allergology, Uppsala University, SE-75185, Uppsala, Sweden; [4]Unit of Environmental Cancer Epidemiology, International Agency for Research on Cancer, 69008 Lyon, France; and [5]Department of Epidemiology, Harvard School of Public Health, Boston, MA 02115, USA

**Summary** In the first cohort study of the question we followed 92 986 (42 663 men and 50 323 women) adult patients hospitalized for asthma in Sweden from 1965 to 1994 for an average of 8.5 years to evaluate their risk of oesophageal and gastric cardia adenocarcinoma. Standardized incidence ratio (SIR) adjusted for gender, age and calendar year was used to estimate relative risk, using the Swedish nationwide cancer incidence rates as reference. Asthmatic patients overall had a moderately elevated risk for oesophageal adenocarcinoma (SIR = 1.5, 95% confidence interval CI, 0.9–2.5) and gastric cardia cancer (SIR = 1.4, 95% CI, 1.0–1.9). However, the excess risks were largely confined to asthmatic patients who also had a discharge record of gastro-oesophageal reflux (SIR = 7.5, 95% CI, 1.6–22.0 and SIR = 7.1, 95% CI, 3.1–14.0, respectively). No significant excess risk for oesophageal squamous-cell carcinoma or distal stomach cancer was observed. In conclusion, asthma is associated with a moderately elevated risk of developing oesophageal or gastric cardia adenocarcinoma. Special clinical vigilance vis-à-vis gastro-esophageal cancers seems unwarranted in asthmatic patients, but may be appropriate in those with clinically manifest gastro-oesophageal reflux. © 2001 Cancer Research Campaign http://www.bjcancer.com

**Keywords**: asthma; gastro-oesophageal reflux; adenocarcinoma of the oesophagus and gastric cardia

A rapid rise in the incidence of oesophageal adenocarcinoma in western societies (Devesa et al, 1998; Hansson et al, 1993) has coincided with an increase in the prevalence of asthma (Sears, 1997). Some common asthma medications, including theophylline and β agonists, relax the lower oesophageal sphincter and facilitate gastro-oesophageal reflux, one of the strongest known risk factors for oesophageal adenocarcinoma (Chow et al, 1995; Farrow et al, 2000; Lagergren et al, 1999a). Indeed, use of these drugs is associated with an up to three-fold increased risk of oesophageal adenocarcinoma (Lagergren et al, 2000a; Vaughan et al, 1998). The fact that asthmatic patients have a higher prevalence of gastro-oesophageal reflux than the general population (Sontag et al, 1990, 1992) raises the question whether the risk of oesophageal adenocarcinoma is such as to warrant special clinical attention or screening. Further, the more common gastric cardia cancers are also associated with gastro-oesophageal reflux, albeit more weakly (Lagergren et al, 1999a), but previous studies failed to find a link with asthma medications (Lagergren et al, 2000a; Vaughan et al, 1998). Thus, the relationship between asthma and cardia cancer remains an open question. We therefore conducted a large retrospective cohort study to quantify the risk of oesophageal and gastric cardia adenocarcinoma, and as a comparison, the risk of oesophageal squamous-cell carcinoma and distal stomach cancer, in adult asthmatic patients, using data from several nationwide registers in Sweden.

*Received 20 June 2001*
*Revised 14 August 2001*
*Accepted 22 August 2001*

*Correspondence to:* W Ye

### METHODS

### Study cohort

We used the Swedish Inpatient Register, founded in 1964–65 by the National Board of Health and Welfare, to create a cohort of adult asthmatic patients. Each record in this register corresponds to one in-hospital episode and contains, in addition to a unique national registration number assigned to each Swedish resident, administrative and medical data such as hospital department, surgical codes, and up to six discharge diagnoses. During our study period, the diagnoses were coded according to the seventh revision of the International Classification of Diseases (ICD-7) through 1968, the eighth revision until 1987 (ICD-8), and the ninth revision thereafter (ICD-9). The number of hospitals delivering data to the register has increased steadily: the register covered 60% of the Swedish population in 1969, 75% in 1978, and 85% by the end of 1983 (Nyren et al, 1995). From 1987, the register attained complete nationwide coverage. We identified a total of 118, 106 unique national registration numbers that were registered at least once with a discharge diagnosis of asthma (ICD-7 = 241.00, 241.01; ICD-8 = 493.00, 493.09; ICD-9 = 493A, 493B, 493X) at age 20 or older between 1965 and 1994.

### Follow-up

Record linkage of the study cohort to the nationwide Register of Causes of Death allowed us to identify the date of death among those deceased through 1995. Corresponding linkage to the Emigration Register identified dates of emigration. The National Swedish Cancer Register, founded in 1958 and close to 98% complete (Mattsson et al, 1985), was used to ascertain all incident cancers from the start of follow-up until December 31, 1995. The Cancer Register coded malignant neoplasms according to the ICD-7 classification during the entire period of study. Through additional linkage to the Total Population Register, we excluded 4243 records, because their national registration numbers could not be found in any of the aforementioned registers and were deemed incorrect numbers. Since only first primary cancers were eligible for study, we also excluded 8157 patients with prevalent cancers at the time of entry. We further excluded 3017 patients who died during the index hospitalization and 161 patients with inconsistencies uncovered during record linkages. To minimize the impact of possible selection bias, we excluded the first year of observation after the index hospitalization, including 9542 patients who reached any end-point (see below) during the first year. Such bias arises if asthmatic patients with a yet undiagnosed cancer are more likely to be hospitalized than asthmatic patients without a subclinical cancer. These 'missed' cases are most likely to become diagnosed during the first year of follow-up. Finally, a total of 92 986 patients, 42 663 men and 50 323 women, remained for further analysis.

### Statistical analysis

Follow-up began from the second year following discharge after the first recorded (index) hospitalization for asthma, until the occurrence of the first cancer diagnosis, emigration, death, or the end of the observation period (December 31, 1995), whichever came first. Since a separate code for gastric cancer in the cardia was introduced first in 1969, cases and person-years in the stomach cancer analyses were accumulated from 1970, 1 year after the coding change to ensure better data quality. To avoid possible ascertainment bias associated with differential autopsy rates between asthmatic patients and the general population, we excluded cancers found incidentally at autopsy. Relative risk of cancer in the cohort was estimated as the standardized incidence ratio (SIR), i.e., the ratio of the observed number of cancers to that expected. The expected number of cancers were calculated by multiplying the number of person-years observed in age- (in 5-year groups), gender-, and calendar year strata in the cohort by stratum-specific cancer incidence rates for the entire Swedish population excluding cancers found incidentally at autopsy. The 95% confidence interval (CI) of the SIR was calculated by the exact method on the assumption that the observed number of cases follows a Poisson distribution (Breslow and Day, 1987).

We did not have clinical data about severity, duration of asthma before the first hospitalization, or treatment. However, we were able to identify co-existing diseases, namely chronic bronchitis/emphysema and gastro-oesophageal reflux diseases (oesophagitis, hiatal hernia, and heart-burn symptoms), diagnosed before/at entry or during follow-up, and use these variables as indicators for severity of asthma or exposure to other risk factors. Stratified analyses were performed on presence or absence of co-existing disease, allocating person-years before the onset of co-existing diseases to the comorbidity-negative strata. We also stratified the analyses by selected cohort characteristics that may influence the results, including age or calendar period at cohort entry, and follow-up duration.

### RESULTS

Some details of the study cohort are listed in Table 1. On average, asthmatic patients were followed up for 8.5 years, resulting in a total of 698 688 (622 483 for stomach cancer analysis) observed person-years. About 4 percent of asthmatic patients had ever been hospitalized for gastro-oesophageal reflux diseases before or during follow-up. The corresponding figure for chronic bronchitis/emphysema was 21.7%. During the period 1–30 years following the initial hospitalization for asthma, we observed 17 cases of oesophageal adenocarcinoma (mean age at diagnosis, 68.6 years) and 43 cases of cardia cancer (mean age at diagnosis, 71.4 years).

**Table 1** Characteristics of the cohort of adult asthmatic patients, 1965–94, Sweden

| Characteristic | |
|---|---|
| Number of patients | 92 986 |
| Distribution by gender (%) | |
| Male | 42 663 (45.9) |
| Female | 50 323 (54.1) |
| Distribution by age at entry, year (%) | |
| 20–49 | 25 814 (27.8) |
| 50–59 | 16 174 (17.4) |
| 60–69 | 22 483 (24.2) |
| 70 + | 28 515 (30.7) |
| Average age at entry, years | 59.1 |
| Distribution by calendar year at entry (%) | |
| 65–74 | 16 036 (17.2) |
| 75–84 | 31 225 (33.6) |
| 85–94 | 45 725 (49.2) |
| Average calendar year at entry | 1983 |
| Distribution by duration of follow-up, years (%) | |
| 1–4 | 35 703 (38.4) |
| 5–9 | 26 503 (28.5) |
| 10 + | 30 780 (33.1) |

### Oesophageal cancer

Compared with the age- and gender-matched general population, there was a 50% excess risk for oesophageal adenocarcinoma (SIR = 1.5, 95% CI 0.9–2.5) (Table 2). Stratified analysis by gender revealed a higher relative risk among men than among women, though the difference was not statistically significant. When subdivided by follow-up duration, a decreased relative risk was found in the first 4 years of follow-up. A more than 2-fold risk was detected for those followed-up 5 years or more (SIR = 2.4, 95% CI 1.4–4.0). Asthmatic patients who had ever been hospitalized for gastro-oesophageal reflux diseases had a higher point estimate (SIR = 7.5, 95% CI 0.7–22.0), while only a non-significant 30% excess of relative risk was observed in the patients without documented gastro-oesophageal reflux diseases (*P* value for difference between two SIRs $< 0.01$). Patients recorded with co-existing chronic bronchitis/emphysema had a higher relative risk than those without, although the difference was not significant (Table 2). The relative risk tended to increase with increasing calendar year of entry into the cohort, or with decreasing age at entry, although none of these trends was statistically significant (data not shown).

No significant excess risk was detected for squamous-cell carcinoma among asthmatic patients (Table 2). The pattern of risk did not differ materially by follow-up duration, with the relative risk below unity after 10 years of follow-up (SIR = 0.8, 95% CI 0.3–1.4). The risks also did not differ noticeably whether or not a concomitant condition was present (Table 2). In addition, the risk

 © 2001 Cancer Research Campaign

**Table 2** Standardized incidence ratios (SIRs) and 95% confidence intervals (CIs) for cancers of the oesophagus among adult asthmatic patients, 1965–94, Sweden

| | Adenocarcinoma of the oesophagus | | | Squamous-cell carcinoma of the oesophagus | | |
|---|---|---|---|---|---|---|
| **Characteristic** | **Obs*** | **SIR** | **95% CI** | **Obs*** | **SIR** | **95% CI** |
| Total | 17 | 1.5 | 0.9–2.5 | 46 | 1.1 | 0.8–1.4 |
| Gender | | | | | | |
| Male | 15 | 1.7 | 1.0–2.8 | 37 | 1.3 | 0.9–1.7 |
| Female | 2 | 0.9 | 0.1–3.1 | 9 | 0.7 | 0.3–1.3 |
| Follow-up, year | | | | | | |
| 1–4 | 1 | 0.2 | 0.01–1.3 | 19 | 1.1 | 0.6–1.7 |
| 5–9 | 8 | 2.4 | 1.0–4.7 | 18 | 1.4 | 0.8–2.2 |
| ≥ 10 | 8 | 2.5 | 1.1–4.9 | 9 | 0.8 | 0.3–1.4 |
| Gastro-oesophageal reflux diseases† | | | | | | |
| No | 14 | 1.3 | 0.7–2.2 | 45 | 1.1 | 0.8–1.5 |
| Yes | 3 | 7.5 | 1.6–22.0 | 1 | 0.7 | 0.02–3.9 |
| Chronic bronchitis or emphysema† | | | | | | |
| No | 10 | 1.2 | 0.6–2.2 | 35 | 1.1 | 0.7–1.5 |
| Yes | 7 | 2.7 | 1.1–5.6 | 11 | 1.2 | 0.6–2.1 |

*Observed number of cancer cases.
†Person-years before the onset of co-existing diseases were allocated to the comorbidity-negative stratum.

**Table 3** Standardized incidence ratios (SIRs) and 95% confidence intervals (CIs) for cancers of the stomach among adult asthmatic patients, 1970–94, Sweden

| | Cancer of the gastric cardia | | | Cancer of the stomach other than cardia | | |
|---|---|---|---|---|---|---|
| **Characteristic** | **Obs*** | **SIR** | **95% CI** | **Obs*** | **SIR** | **95% CI** |
| Total | 43 | 1.4 | 1.0–1.9 | 219 | 0.9 | 0.8–1.1 |
| Gender | | | | | | |
| Male | 28 | 1.2 | 0.8–1.8 | 136 | 0.9 | 0.8–1.1 |
| Female | 15 | 2.0 | 1.1–3.3 | 83 | 0.9 | 0.7–1.1 |
| Follow-up, year | | | | | | |
| 1–4 | 13 | 1.0 | 0.5–1.7 | 113 | 1.0 | 0.8–1.2 |
| 5–9 | 19 | 2.0 | 1.2–3.1 | 73 | 1.0 | 0.8–1.2 |
| ≥ 10 | 11 | 1.4 | 0.7–2.5 | 33 | 0.6 | 0.4–0.9 |
| Gastro-oesophageal reflux diseases† | | | | | | |
| No | 35 | 1.2 | 0.8–1.7 | 214 | 0.9 | 0.8–1.1 |
| Yes | 8 | 7.1 | 3.1–14.0 | 5 | 0.6 | 0.2–1.5 |
| Chronic bronchitis or emphysema† | | | | | | |
| No | 27 | 1.2 | 0.8–1.7 | 148 | 0.8 | 0.7–0.9 |
| Yes | 16 | 2.2 | 1.3–3.6 | 71 | 1.3 | 1.0–1.7 |

*Observed number of cancer cases.
†Person-years before the onset of co-existing diseases were allocated to the comorbidity-negative stratum.

pattern did not change substantially across age or calendar period at cohort entry (data not shown).

### Stomach cancer

A marginally significant 40% excess risk of gastric cardia cancer was observed (SIR = 1.4, 95% CI 1.0–1.9) in asthmatic patients, with no consistent change with follow-up duration (Table 3). The relative risk was higher among women than among men, though the difference was not statistically significant. Stratification according to comorbidity showed relative risks close to unity in those without recorded co-existing diseases (Table 3). The excess risk increased with increasing calendar year of entry into the cohort, or with decreasing age at cohort entry, although none of these trends was statistically significant (data not shown).

There was no increased risk for cancer of the distal stomach. In fact, a significant reduction in risk was observed after 10 or more years of follow-up. No obvious difference was noted by comorbidity status (Table 3).

### DISCUSSION

We found a 50% increased relative risk for oesophageal adenocarcinoma among asthmatic patients, consistent with the hypothesized link via gastro-oesophageal reflux. Also observed was a marginally significant 40% excess risk of gastric cardia cancer. There was no excess risk of oesophageal squamous-cell carcinoma or stomach cancer distal to the gastric cardia.

Gastro-oesophageal reflux has been associated with substantial increase in risk of oesophageal adenocarcinoma (Chow et al, 1995; Farrow et al, 2000; Lagergren et al, 1999a). The prevalence of gastro-oesophageal reflux symptoms is reportedly higher in asthmatic patients than in subjects without asthma (Sontag et al, 1990, 1992), though some may have clinically silent reflux (Irwin et al,

© 2001 Cancer Research Campaign

1993). These observations are supported by clinical evidence among asthmatic patients indicating a high prevalence of oesophagitis (Sontag et al, 1992), decreased oesophageal sphincter pressure (Kjellen et al, 1981; Sontag et al, 1990), and increased acid contact time measured through ambulatory 24-hour oesophageal pH monitoring (Harding et al, 1999; Vincent et al, 1997). Nevertheless, the relationship between gastro-oesophageal reflux and asthma is not well understood. While gastro-oesophageal reflux may be a trigger for asthma and medical or surgical treatment of reflux can improve asthma outcomes (Field et al, 1999; Field and Sutherland, 1998), medications for asthma can cause or worsen gastro-oesophageal reflux. Recent epidemiological studies have linked asthma medications, including theophylline and β-agonists, to an increased risk of oesophageal adenocarcinoma (Lagergren et al, 2000a; Vaughan et al, 1998). However, in epidemiological studies based on self-reported medication and medical histories it is difficult to separate the effects of asthma drugs from those of possible underlying reflux associated with asthma. Nevertheless, our results suggest that the absolute risk of oesophageal adenocarcinoma among asthma patients is small.

Gastro-oesophageal reflux is associated with a moderately increased risk of gastric cardia adenocarcinoma (Chow et al, 1995; Lagergren et al, 1999a), but of the two studies of the association between asthma drug treatment and gastro-oesophageal adenocarcinoma risk, neither found any excess risk of cardia cancer associated with such medication (Lagergren et al, 2000a; Vaughan et al, 1998). We observed an elevated relative risk for gastric cardia cancer among asthmatic patients, of similar magnitude to that for oesophageal adenocarcinoma. Smoking, a risk factor for these cancers (Gammon et al, 1997; Lagergren et al, 2000b) and an aggravating factor of adult asthma (Floreani and Rennard, 1999), may not be an explanation, since we did not observe any excess of oesophageal squamous-cell carcinoma, which is more strongly linked to smoking (Gammon et al, 1997; Lagergren et al, 2000b). Obesity is another possible confounder (Camargo et al, 1999; Chow et al, 1998; Lagergren et al, 1999b). Unfortunately, the restrictive use of the obesity diagnosis in overweight patients hospitalized for other reasons than their weight problem prevented us from effectively controlling for this condition.

To the best of our knowledge, this is the first cohort study to examine the risk of adenocarcinomas of the oesophagus and gastric cardia in subjects with asthma. The design of the present study has the strength of reducing potential recall and selection biases. Further, the high quality of Swedish national registers ensures near-complete follow-up, and high accuracy of our outcome information with 90–100% of the oesophageal cancers being histologically confirmed (Centre for epidemiology, the National Board of Health and Welfare, 1975, 1985, 1995). However, some limitations should also be noted. First, data on asthma severity, duration before index hospitalization or treatment is lacking. Asthma is a common disease, and those who were hospitalized are likely to have had more severe forms or more co-existing diseases. Thus, our estimates may not be representative of asthma as a whole and, if severity increases cancer risk, we may have overestimated the risk of gastro-oesophageal adenocarcinomas. On the other hand, we used the general population as reference, in which around 5% have asthma (Lundback et al, 1993; Montnemery et al, 1998), which would lead to some underestimation of the true association. Finally, despite the relatively large cohort size, the number of cancer cases was small, yielding limited statistical power for detailed analysis.

In conclusion, asthmatic patients are at moderately elevated risk of developing oesophageal or gastric cardia adenocarcinoma. The most plausible mechanism is via gastro-oesophageal reflux. The absolute excess risk for these two types of adenocarcinomas was small so that management changes seem unjustified. However, the possibility of oesophageal and gastric cardia adenocarcinoma development in asthmatic patients with clinically manifest gastro-oesophageal reflux should be kept in mind.

### ACKNOWLEDGEMENT

This study was supported by a grant 4279-B99-01XAB from Swedish Cancer Society.

### REFERENCE

Breslow NE and Day NE (1987) The design and analysis of cohort studies. In: *Statistical methods in cancer research*, Vol 2. IARC Scientific Publications No. 82. International Agency for Research on Cancer: Lyon

Camargo CA Jr, Weiss ST, Zhang S, Willett WC and Speizer FE (1999) Prospective study of body mass index, weight change, and risk of adult-onset asthma in women. *Arch Intern Med*; **159**: 2582–2588

Centre for epidemiology, the National Board of Health and Welfare (1975) *Cancer Incidence in Sweden 1975*. Stockholm, Sweden

Centre for epidemiology, the National Board of Health and Welfare (1985) *Cancer Incidence in Sweden 1985*. Stockholm, Sweden

Centre for epidemiology, the National Board of Health and Welfare (1995) *Cancer Incidence in Sweden 1995*. Stockholm, Sweden

Chow WH, Finkle WD, McLaughlin JK, Frankl H, Ziel HK and Fraumeni JF Jr (1995) The relation of gastroesophageal reflux disease and its treatment to adenocarcinomas of the esophagus and gastric cardia. *JAMA* **274**: 474–477

Chow WH, Blot WJ Vaughan TL Risch HA, Gammon MD, Stanford JL, Dubrow R, Schoenberg JB, Mayne ST, Farrow DC, Ahsan H, West AB, Rotterdam H, Niwa S and Fraumeni JF Jr (1998) Body mass index and risk of adenocarcinomas of the esophagus and gastric cardia. *J Natl Cancer Inst* **90**: 150–155

Devesa SS, Blot WJ and Fraumeni JF Jr (1998) Changing patterns in the incidence of esophageal and gastric carcinoma in the United States. *Cancer* **83**: 2049–2053

Farrow DC, Vaughan TL, Sweeney C, Gammon MD, Chow WH, Risch HA, Stanford JL, Hansten PD, Mayne ST, Schoenberg JB, Rotterdam H, Ahsan H, West AB, Dubrow R, Fraumeni JF, Jr and Blot WJ (2000) Gastroesophageal reflux disease, use of H2 receptor antagonists, and risk of esophageal and gastric cancer. *Cancer Causes Control* **11**: 231–238

Field SK, Underwood M, Brant R and Cowie RL (1996) Prevalence of gastroesophageal reflux symptoms in asthma. *Chest* **109**: 316–322

Field SK and Sutherland LR (1998) Does medical antireflux therapy improve asthma in asthmatics with gastroesophageal reflux?: a critical review of the literature. *Chest* **114**: 275–283

Field SK, Gelfand GA and McFadden SD (1999) The effects of antireflux surgery on asthmatics with gastroesophageal reflux. *Chest* **116**: 766–774

Floreani AA and Rennard SI (1999) The role of cigarette smoke in the pathogenesis of asthma and as a trigger for acute symptoms. *Curr Opin Pulm Med* **5**: 38–46

Gammon MD, Schoenberg JB, Ahsan H, Risch HA, Vaughan TL, Chow WH, Rotterdam H, West AB, Dubrow R, Stanford JL, Mayne ST, Farrow DC, Niwa S, Blot WJ and Fraumeni JF Jr (1997) Tobacco, alcohol, and socioeconomic status and adenocarcinomas of the esophagus and gastric cardia. *J Natl Cancer Inst* **89**: 1277–1284

Hansson LE, Sparen P and Nyren O (1993) Increasing incidence of both major histological types of esophageal carcinomas among men in Sweden. *Int J Cancer* **54**: 402–407

Harding SM, Guzzo MR and Richter JE (1999) 24-h esophageal pH testing in asthmatics: respiratory symptom correlation with esophageal acid events. *Chest* **115**: 654–659

Irwin RS, Curley FJ and French CL (1993) Difficult-to-control asthma. Contributing factors and outcome of a systematic management protocol. *Chest* **103**: 1662–1669

Kjellen G, Brundin A, Tibbling L and Wranne B (1981) Oesophageal function in asthmatics. *Eur J Respir Dis* **62**: 87–94

 *© 2001 Cancer Research Campaign*

Lagergren J, Bergstrom R, Lindgren A and Nyren O (1999a) Symptomatic gastroesophageal reflux as a risk factor for esophageal adenocarcinoma. *N Engl J Med* **340**: 825–831
Lagergren J, Bergstrom R and Nyren O (1999b). Association between body mass and adenocarcinoma of the esophagus and gastric cardia. *Ann Intern Med* **130**: 883–890
Lagergren J, Bergstrom R, Adami HO and Nyren O (2000a) Association between Medications That Relax the Lower Esophageal Sphincter and Risk for Esophageal Adenocarcinoma. *Ann Intern Med* **133**: 165–175
Lagergren J, Bergstrom R, Lindgren A and Nyren O (2000b) The role of tobacco, snuff and alcohol use in the aetiology of cancer of the oesophagus and gastric cardia. *Int J Cancer* **85**: 340–346
Lundback B, Stjernberg N, Nystrom L, Lundback K, Lindstrom M and Rosenhall L (1993) An interview study to estimate prevalence of asthma and chronic bronchitis. The obstructive lung disease in northern Sweden study. *Eur J Epidemiol* **9**: 123–133
Mattsson B, Rutqvist LE and Wallgren A (1985) Undernotification of diagnosed cancer cases to the Stockholm Cancer Registry. *Int J Epidemiol* **14**: 64–69
Montnemery P, Adelroth E, Heuman K, Johannisson A, Johansson SA, Lindholm LH, Lundback B and Lofdahl CG (1998) Prevalence of obstructive lung diseases and respiratory symptoms in southern Sweden. *Respir Med* **92**: 1337–1345
Nyren O, McLaughlin JK, Gridley G, Ekbom A, Johnell O, Fraumeni JJ and Adami HO (1995) Cancer risk after hip replacement with metal implants: a population-based cohort study in Sweden. *J Natl Cancer Inst* **87**: 28–33
Sears MR (1997) Descriptive epidemiology of asthma. *Lancet* **350 (Suppl 2)**: SII1–SII4
Sontag SJ, O'Connell S, Khandelwal S, Miller T, Nemchausky B, Schnell TG and Serlovsky R (1990) Most asthmatics have gastroesophageal reflux with or without bronchodilator therapy. *Gastroenterology* **99**: 613–620
Sontag SJ, Schnell TG, Miller TQ, Khandelwal S, O'Connell S, Chejfec G, Greenlee H, Seidel UJ and Brand L (1992) Prevalence of oesophagitis in asthmatics. *Gut* **33**: 872–876
Vaughan TL, Farrow DC, Hansten PD, Chow WH, Gammon MD, Risch HA, Stanford JL, Schoenberg JB, Mayne ST, Rotterdam H, Dubrow R, Ahsan H, West AB, Blot WJ and Fraumeni JF Jr (1998) Risk of esophageal and gastric adenocarcinomas in relation to use of calcium channel blockers, asthma drugs, and other medications that promote gastroesophageal reflux. *Cancer Epidemiol Biomarkers Prev* **7**: 749–756
Vincent D, Cohen-Jonathan AM, Leport J, Merrouche M, Geronimi A, Pradalier A and Soule JC (1997) Gastro-oesophageal reflux prevalence and relationship with bronchial reactivity in asthma. *Eur Respir J* **10**: 2255–2259

*© 2001 Cancer Research Campaign*
